# Hyperacute iris neovascularization following cataract surgery

**DOI:** 10.1097/MD.0000000000029356

**Published:** 2022-07-08

**Authors:** Ha Eun Sim, Je Hyung Hwang

**Affiliations:** a Department of Ophthalmology, Sanggye Paik Hospital, Inje University of Korea, College of Medicine, Seoul, Republic of Korea.

**Keywords:** cataract surgery, hemi-CRVO, iris neovascularization

## Abstract

**Rationale:**

We describe a case of acute neovascularization of the iris after uneventful cataract surgery.

**Patient concerns:**

A 78-year-old man visited our clinic for cataract surgery and glaucoma management.

**Diagnoses:**

The patient underwent bilateral laser iridotomy 4 years ago. On ocular examination, the best-corrected visual acuity was no light perception in the right eye and 20/100 in the left eye. We observed pseudophakic bullous keratopathy in the right eye and cataracts and hemicentral retinal vein occlusion (CRVO) in the left eye.

**Interventions:**

The patient underwent cataract surgery in the left eye without complications.

**Outcomes:**

The day after surgery we observed 360° of neovascularization in the iris and aggravated hemi-CRVO with macular edema. Therefore, we administered intravitreal bevacizumab in the left eye, after which the iris neovascularization and macular edema improved.

**Lessons:**

Cataract surgery can rapidly aggravate hemi-CRVO and cause iris neovascularization, which is responsive to bevacizumab.

## 1. Introduction

Age-related cataracts are a major cause of visual impairment and blindness worldwide, with cataract extraction being the most frequently performed eye surgery.^[1]^ With continued development of surgical methods, the safety and efficacy of cataract surgery have steadily improved; however, complications may still occur.

Central retinal vein occlusion (CRVO) and branch retinal vein occlusion are major causes of vision loss.^[2,3]^ Retinal vein occlusion is the second most common cause of retinal vascular disease following diabetic retinopathy.^[2]^ There have been various studies of ocular neovascularization in CRVO and hemi-CRVO.^[3–5]^ Neovascularization of the iris (NVI) is a rare complication observed in retinal ischemic diseases, including CRVO. NVI can advance to the trabecular meshwork and obstruct the aqueous humor outlet. Consequently, neovascular glaucoma can lead to blindness.^[6]^

Herein, we report a case of acute-onset iris neovascularization and aggravated hemi-CRVO with macular edema following uneventful cataract surgery.

## 2. Case report

A 78-year-old man visited our clinic for cataract surgery and glaucoma management. His medical history was positive for well-controlled hypertension and Parkinson disease. He underwent bilateral laser iridotomy for narrow angles 4 years ago and cataract surgery in the right eye. He was diagnosed with open-angle glaucoma in the left eye after his symptoms were relieved by administering a brinzolamide/timolol (Elazop; Alcon, Geneva, Switzerland) antiglaucoma eye drop.

**Figure 1.**
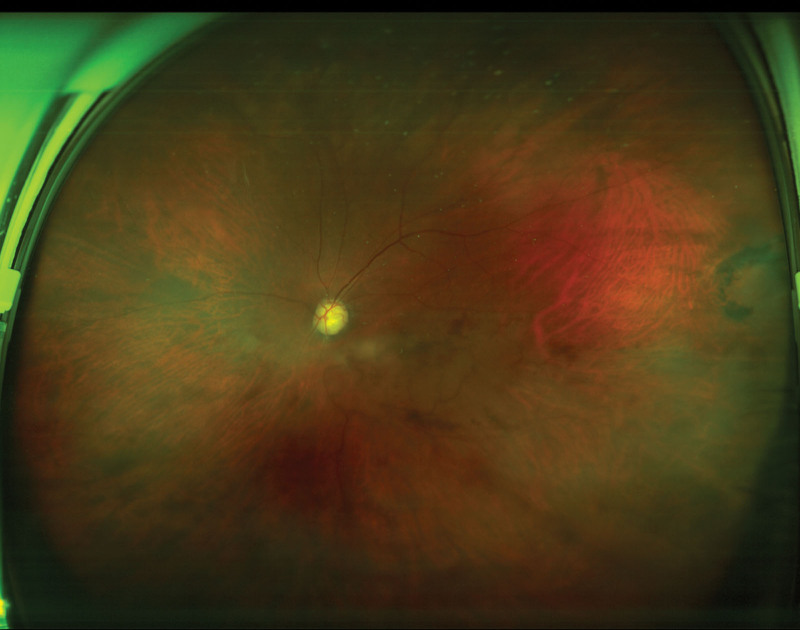
Fundus examination of the hemicentral retinal vein occlusion before the cataract surgery.

On ocular examination, his best-corrected visual acuity (BCVA) was no light perception in the right eye and 20/100 (+6.00 Dsph;-3.00 A90) in the left eye. Using a noncontact tonometer, the intraocular pressure was 48/13 mm Hg. On slit-lamp examination, pseudophakic bullous keratopathy was observed in the right eye, while nonspecific findings except cataract were seen in the left eye. On fundus examination, the posterior pole was not observed because of dim vision in the right eye, while hemi-CRVO with a glaucomatous optic disc was observed in the left eye (Fig. 1). Optical coherence tomography did not reveal macular edema in the left eye. The anterior chamber depth was 2.66 mm on ocular biometry, and the axial length was 23.15 mm in the left eye.

We decided to perform cataract surgery after considering its risks and benefits. Phacoemulsification with intraocular lens implantation was performed under topical anesthesia using the Centurion Vision System (Alcon Laboratories, Inc., Geneva, Switzerland) via a 2.3-mm clear corneal incision. No complications were observed.

**Figure 2.**
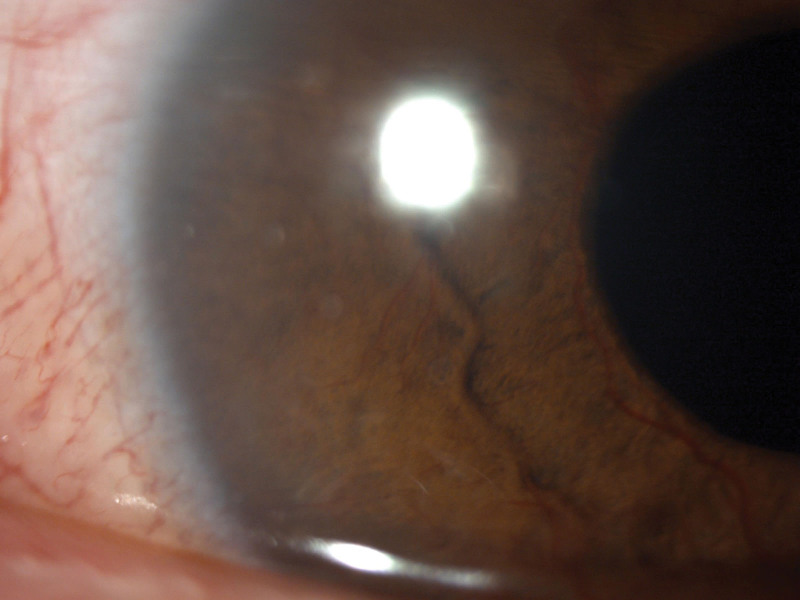
Slit-lamp examination of the neovascularization of the iris after the cataract surgery.

**Figure 3.**
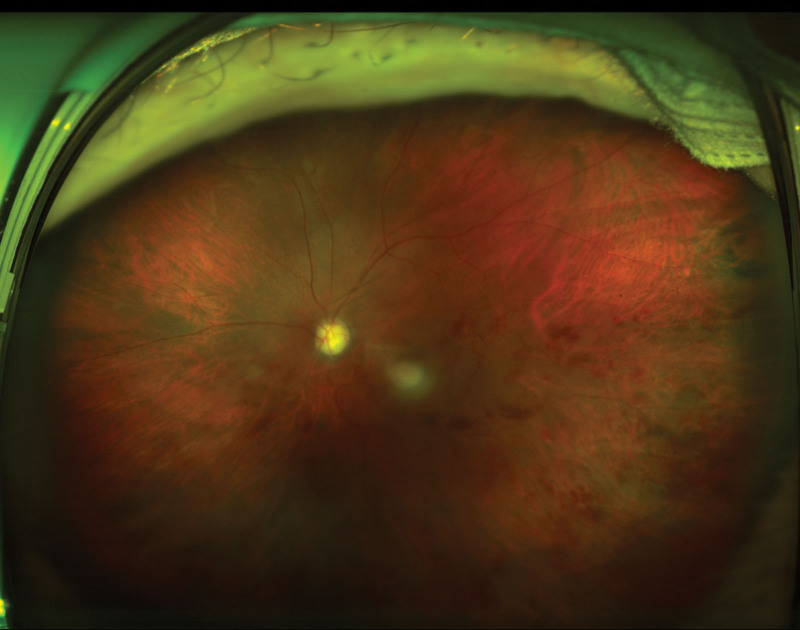
Fundus examination of the aggravated hemicentral retinal vein occlusion after the cataract surgery.

One day after surgery, his BCVA was 20/400 in the operated eye, and the intraocular pressure was 16 mmHg on a noncontact tonometer. On slit-lamp examination, corneal edema, 2+ cells in the anterior chamber, and 360° NVI were observed (Fig. 2). Fundus examination of the left eye revealed aggravated hemi-CRVO and macular edema (Fig. 3) for which intravitreal 0.05 cc bevacizumab (Avastin) was administered. On postoperative day 2, the BCVA and intraocular pressure in the left eye improved to 20/63 and 16 mm Hg, respectively. On slit-lamp examination, the NVI was observed to have regressed.

Three months after surgery, his BCVA was 20/63 and intraocular pressure 12 mm Hg in the left eye, with no recurrence of NVI. Fundus examination showed no recurrence of macular edema.

## 3. Discussion

This report presents a case of acute-onset NVI and aggravated hemi-CRVO with macular edema following uneventful cataract surgery. Previous studies reported that the cumulative incidences of NVI at 12 and 36 months were 6.1% and 8.5%, respectively, in the CRVO group and 1.3% and 2.4%, respectively, in the branch retinal vein occlusion group.^[7]^

Hayreh et al^[8]^ reported that the incidence of NVI at 12 months was 12% in patients with ischemic hemi-CRVO and 0% in patients with nonischemic hemi-CRVO. Other studies have reported a similar incidence of NVI in nonischemic and ischemic hemi-CRVO.^[3,9]^ We did not perform preoperative fluorescein angiography because of the relatively dim vision caused by the cataract. We planned to perform it after cataract surgery. However, the development of NVI strongly suggests the possibility of ischemic hemi-CRVO.

Several risk factors—including systemic hypertension, dyslipidemia, and ocular conditions—are associated with retinal vein occlusion.^[10,11]^ Increased intraocular pressure and glaucoma are associated with the risk of retinal vein occlusion.^[12,13]^ Our patient’s risk factors included systemic hypertension and glaucoma.

Cataract surgery is the presumed trigger for retinal vein occlusion. Phacoemulsification may increase the intraocular pressure, leading to retinal artery hypoperfusion.^[14]^ In our case, the increased intraocular pressure during cataract surgery may have aggravated the preexisting hemi-CRVO.

Vascular endothelial growth factor plays an important role in the complications of retinal vein occlusion. The intravitreal administration of bevacizumab improves visual acuity and reduces central retinal thickness.^[15]^ Intravitreal injection of antivascular endothelial growth factor has the potential to inhibit neovascularization not only in the retina but also in the iris. In our case, NVI decreased a day after the intravitreal administration of bevacizumab.

Rodrigues et al^[16]^ described rare cases without neovascularization in the angle, and without the pupil, especially after ischemic CRVO. Because we did not perform gonioscopy, we might have missed preexisting neovascularization of the angle.

In this case, cataract surgery may have been the cause of the acute-onset iris neovascularization and aggravated hemi-CRVO. Therefore, careful examination of the iris and gonioscopy is essential before pupil dilation is performed in patients with preexisting retinal vein occlusion.

### Author contributions

Substantial contributions to the conception or design of the work: H.J.H.

Data collection: H.J.H.

Drafting the work: S.H.E.
